# A Comparative Analysis of the Gene Expression Profiles in the Mammary Glands of Lactating and Nonlactating Mares at the Second Month of Gestation

**DOI:** 10.3390/ani14162319

**Published:** 2024-08-09

**Authors:** Tseweendolmaa Ulaangerel, Min Wang, Bilig Zhao, Minna Yi, Yingchao Shen, Yibeeltu Mengkh, Xin Wen, Manglai Dugarjav, Gerelchimeg Bou

**Affiliations:** 1 Equus Research Center, College of Animal Science, Inner Mongolia Agricultural University, Hohhot 010018, China; cewendclma@126.com (T.U.); wangmin202128@163.com (M.W.); bilig9@163.com (B.Z.); yiminna2020@163.com (M.Y.); shenyingchao2017@163.com (Y.S.); wenxin618@imau.edu.cn (X.W.); dmanglai@163.com (M.D.); 2 Animal Quarantine & Disease Control Center, Darhan-Muminggan Joint County, Baotou 014500, China; 3 Xilingol League Animal Husbandry Work Station, Xilinhot 026099, China; ybeelt@163.com

**Keywords:** mare’s milk, transcriptome sequencing, pregnancy

## Abstract

**Simple Summary:**

To explore molecular regulation related to lactation during pregnancy, the mammary glands of pregnant Mongolian mares during lactation and non-lactation were sequenced and analyzed for differentially expressed genes. We screened milk protein-related genes (*CSN1S1*, *CSN3*, and *LALBA*), genes related to the lipid metabolism process (*DGAT1*, *LEP*, and *LEPR*), and genes closely related to lactation events (*FAT1* and *LTF*), which can be used as candidate genes for selecting lactation traits in Mongolian mares.

**Abstract:**

To investigate molecular regulation involved in lactation during pregnancy, this study focused on the transcriptomic profiles of mammary tissue from lactating and non-lactating Mongolian mares at the second month of gestation. A total of 4197 differentially expressed genes were identified by comparing mammary tissues from pregnant mares at two different states, including 1974 differentially expressed genes such as the milk protein-related genes a-s1-casein (*CSN1S1*), k-casein (*CSN3*), lactalbumin (*LALBA*), and lactoferrin (*LTF*), which were highly expressed in the lactating mares group, and overall, these differentially expressed genes were mainly associated with biological processes such as endoplasmic reticulum protein processing, the Toll-like receptor signaling pathway, steroid biosynthesis, cytokine–cytokine receptor interactions, and amino sugar and nucleotide glycolysis. These findings serve as a foundation for investigating the molecular underpinnings of lactation in pregnant equids.

## 1. Introduction

The development of the horse industry has gone through a long process of change. The use of horses in power-related roles in service and transportation and as tools for combat, grazing, and hunting has been gradually completely replaced by machinery; the modern horse industry has developed in new directions of economy, tourism, recreation, and entertainment while driving the development of other industry chains. The traditional horse industry continues to decline and is in urgent need of transformation and upgrading. To produce greater economic and social benefits, the unique nutritional and health care role of mare’s milk and its related products and the comprehensive development and utilization of the horse industry around the world have become important directions for the development of horse breeding.

Mare’s milk is reportedly consumed regularly by 30 million people around the world, such as in Mongolia and Kazakhstan in Central Asia, the residents of which consume mare’s milk mainly in the form of fermented products and refer to these beverages as *koumiss*, *airag*, or *chigee*; the consumption of mare’s milk is also common in some European countries [1,2]. In addition to the use of this milk as a valuable source of nutrition, it has long been regarded as a medicine in the former Soviet Union and West Asia because of its health-promoting properties [2]. The growing interest in mare’s milk has been driven to a large extent by information about its positive health effects and the similarity of its composition to human milk.

Mare’s milk is rich in nutrients, including proteins, fats, sugars, phosphorus, calcium, vitamin C, and many other components [3]. The fat content of mare’s milk is significantly lower than that of human milk and cow’s milk, but the proportion of unsaturated fatty acids is greater than that of cow’s milk and close to that of human milk, which is effective in preventing hypercholesterolemia and atherosclerosis [4,5]. Moreover, mare’s milk has high levels of whey and casein proteins, with a concentration between those of human milk and cow’s milk, and the ratio and spongy structure of the granular micelles make it physiologically easier to digest than cow’s milk [6,7]. In addition, the whey protein contents of mare’s milk and human milk are similar and higher than that of cow’s milk. Whey proteins are involved in the conversion of glucose and galactose to lactose, which is an important carbohydrate source for foals [7,8]. It has been shown that the calcium-to-phosphorus ratio of mare’s milk (1.6–1.8:1) is more favorable for the normal growth of hatchling skeletons than that of cow’s milk (approximately 1.4:1) and is closer to that of human milk (approximately 1.9:1) [6]. In addition, mare’s milk is used in various fields, such as for the treatment and prevention of tuberculosis and other bacterial infections, due to its high levels of other bioactive substances, such as lysozyme, and antibacterial and antiviral active ingredients, such as lactoferrin [9]. Lactoferrin not only improves the digestion of nutrients by increasing the surface area of the intestinal mucosa but also promotes the establishment of the intestinal microbiota in newborns [10,11]. These characteristics of horse milk have led to a renewed interest in its lactation performance; however, horses are currently in a state of unselected breeding for milk production with untapped milk production potential, and they generally produce far less milk than the predicted optimum.

In production practice, we found that some mares continue to lactate during gestation or even remain in lactation for a long period of time, a phenomenon we believe may be useful for breeding high-yielding Mongolian dairy horse lines. Therefore, we explored the differentially expressed genes and signaling pathways related to lactation traits during pregnancy. In this study, we sequenced the mammary glands of Mongolian mares during gestational lactation and non-lactation and analyzed the differentially expressed genes to identify candidate genes related to lactation in Mongolian mares. The results of this study will lay the foundation for analyzing the molecular mechanism of lactation and understanding the characteristics of equine milk production.

## 2. Materials and Methods

### 2.1. Collection of Equine Mammary Gland Samples

The animal experiments were reviewed and approved by the Animal Protection and Use Committee of the Inner Mongolia Agricultural University. Mammary gland tissues from lactating and non-lactating healthy 6–8-year-old Mongolian mares in the second month of gestation were obtained separately from the slaughterhouse in East Wuzhumu Banner, Xilin Gol, Inner Mongolia Autonomous Region. Half of the breast tissue samples were snap-frozen in liquid nitrogen and stored at −80 °C until analysis. The other half was fixed in paraformaldehyde and stored at −4 °C until analysis.

### 2.2. Histomorphology Examination

The experimental horse mammary tissue was fixed with 4% paraformaldehyde, dehydrated with a graded ethanol series, washed with xylene, embedded in paraffin, sectioned at a thickness of 7 µm, prepared for tissue sectioning, stained with H&E, and finally photographed under a light microscope.

### 2.3. RNA Extraction, Library Preparation, and Sequencing

Total RNA was extracted from tissue samples using the TRIzol method, and the purity and integrity of total RNA were examined using a NanoDrop 2000 spectrophotometer (Thermo Scientific, Waltham, MA, USA) and Agilent 2100 instruments (Agilent, Santa Clara, CA, USA). The libraries were constructed using an Illumina RNA library prep kit (NEB, Ipswich, MA, USA), and after library construction, their quality, concentration, and size were detected using a Qubit 2.0 (Thermo Scientific, MA, USA) and Agilent 2100 bioanalyzer (Agilent, CA, USA). After meeting the expected standards, sequencing was performed with the Illumina HiSeq^TM^ 2500 (Illumina, San Diego, CA, USA) platform.

### 2.4. Quality Control and Alignment

To ensure the quality and credibility of the data, the sequencing data were filtered using SOAPnuke [12] (v2.1.0). The filtered RNA-seq reads were mapped to the horse reference genome (EquCab3.0) using HISAT2 [13]. After quality control, the second-generation clean sequence reads were compared to the reference transcript sequence using Bowtie2 [14]. The comparison results of Bowtie2 were counted using RSEM [15] (V1.3.1) to obtain the number of reads compared to each transcript for each breast tissue sample and converted to gene expression values (fragments per kilobase per million bases, FPKM) [16], and paired-end reads from the same fragments were counted as fragments, which, in turn, determined the expression levels of the genes and transcripts.

### 2.5. Screening of Differentially Expressed Genes (DEGs)

To compare the FPKM density distribution of genes/transcripts from different samples as a whole, we performed a principal component analysis (PCA) of the FPKM values for all samples. Based on the results of expression quantification, we screened the samples for genes and transcripts with significant differences in expression levels at different stages. DESeq2 [17] (V1.22.2) was used for differential expression significance analysis, and the screening thresholds were FDR (false discovery rate) < 0.05 and log2FC (fold change) > 1 or <−1.

### 2.6. Gene Ontology (GO) and Kyoto Encyclopedia of Genes and Genomes (KEGG) Pathway Enrichment Analysis

Gene classification and biological function annotation were performed using the online DAVID database. The symbols of differentially expressed genes between samples (adjusted *p* < 0.05) were submitted to DAVID. GO and KEGG enrichment analyses were performed for differentially expressed, upregulated, and downregulated genes between samples. Horse genes labeled in the GO and KEGG databases were enriched and analyzed as background genes.

### 2.7. Quantitative Real-Time PCR (qPCR)

TRIzol was used to harvest RNA from samples from the lactation and non-lactation groups, and the RNA samples were converted to cDNA using a reverse transcription kit (Prime Script™ RT Master Mix) (Takara, Kyoto, Japan). Using GAPDH as a control, the reaction system (25 µL) was prepared according to the instructions provided for TB Green^®^Premix Ex Taq^TM^ II (Takara, Kyoto, Japan). The data were analyzed by the 2^−∆∆Ct^ method to determine the expression levels of the genes in the lactation and non-lactation groups of Mongolian mares. The parallel experiment included at least three replicates, and the data are expressed as the mean ± SD. The differences between groups were compared with the GraphPad Prism 9.0 software, and the differences were considered significant when *p* < 0.05. The primers are shown in Supplementary Table S3.

### 2.8. Immunohistochemistry

Dehydrated mammary gland specimens from Mongolian mares (n = 3/group) were embedded in paraffin blocks. Cross sections of 7 μm in thickness were obtained from each block using a microtome (RM 2245, Leica, Wetzlar, Germany) and collected on glass slides and air-dried for 30 min. After dilution of the antibodies using Antibody Dilution Buffer according to the antibody instruction manual, primary antibodies against *CSN3* (AF0187, Affinity, MEL, AUS), *FAT1* (DF8943, Affinity), *DGAT1* (DF13368, Affinity), *LEP* (DF8583, Affinity), *LEPR* (DF7139, Affinity), *LTF* (DF8059, Affinity), *CSN1S1* (DF13271, Affinity), and *LALBA* (A6233, ABclonal, Woburn, MA, USA) were incubated with the slides overnight at 4 °C. After the slides were rinsed in PBS for 5 min, the slides were then incubated dropwise with biotin-labeled secondary antibody for 10 min at room temperature. Finally, a DAB horseradish peroxidase colorimetric kit (DA1015, Solarbio, Beijing, China) was used for color development, and images were captured with a microscope imaging system (Zeiss, Oberkochen, Germany).

### 2.9. Statistical Analysis

To assess significant differences in gene expression across gestations, two-way ANOVA was used to assess probability (*p*) values. Differences were considered significant when *p* < 0.05.

## 3. Results

### 3.1. Histomorphometric Analysis of Horse Mammary Glands

**Figure 1:**
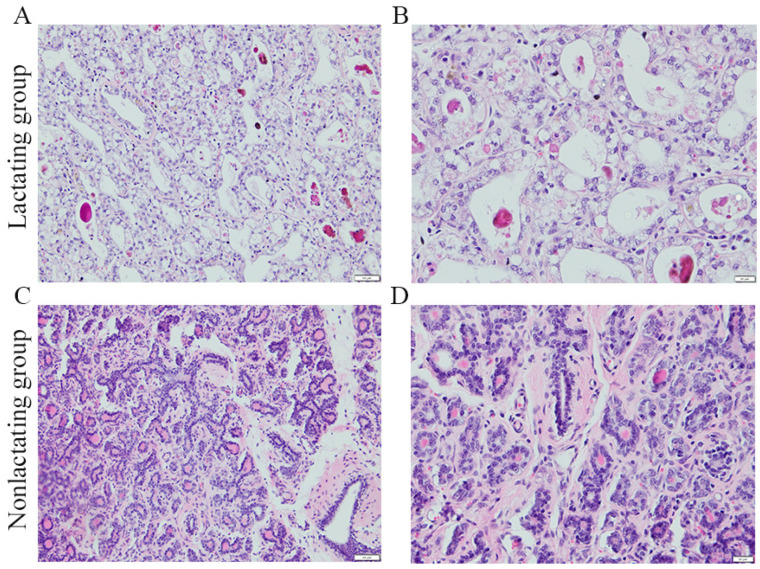
Immunohistochemical staining of mammary gland tissues from lactating and non-lactating mares. (**A**) Lactating status; 20 µm. (**B**) Lactating status; 50 µm. (**C**) Non-lactating status; 20 µm. (**D**) Non-lactating status; 50 µm.

We first studied the morphological and microscopic features of the mammary glands by performing HE staining of the paraffin sections of different tissue samples. The morphological differences in the mammary glands in different lactation states at the second month of gestation are shown in Figure 1. The lobules of the mammary glands in the lactating state were filled with a large number of mature alveolar structures, and the ducts were markedly thickened (Figure 1A,B). In the non-lactating state, the mammary glands were characterized by the presence of large amounts of fat and connective tissue, and the alveoli were smaller and fewer in number (Figure 1C,D). These results indicate that there are significant differences in the mammary tissue morphology between lactating and non-lactating mares.

### 3.2. Basic Statistical Analysis Results of Sequencing Data

To analyze the gene expression profiles in the non-lactating and lactating groups during pregnancy, we sequenced six RNA libraries from Mongolian horse mammary tissues during these two periods, and the original sequence reads were quality-controlled and filtered by the sequencing platform. After removing the contaminated and low-quality reads, the total number of clean reads obtained was 386,339,097, and the clean base content of each sample reached 16,734,242,700 bp or more. The percentage of Q20 bases was greater than 97.7%, the percentage of Q30 bases was greater than 93.4%, and the total GC content after filtration ranged from 41.3.8% to 46.6%, which is close to 50% (Supplementary Table S1). The sequencing libraries generated from the gestational non-lactating and lactating groups had comparison rates of ≥95.95% with the reference genome (Supplementary Table S2).

### 3.3. Differential Expression Analysis Results

**Figure 2:**
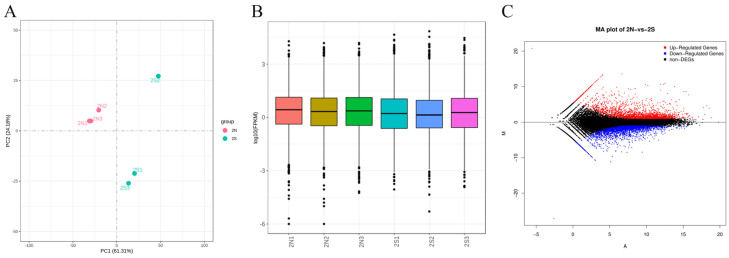
A quality assessment and basic statistics of the sequencing data. (**A**) A principal component analysis of the mammary gland tissue samples. (**B**) Boxplots of the relative log expression of genes in the mammary gland tissue samples. (**C**) Differential genes in the lactating and non-lactating groups during pregnancy. 2N1, 2N2, 2N3: non-lactation group; 2S1, 2S2, 2S3: lactation group.

We analyzed all samples using a principal component analysis. The samples within the gestational lactating and non-lactating groups were clustered with each other, the similarity of the expression patterns between samples was high, and the differences between the groups were large with clear subgrouping (Figure 2A). A comparison of the FPKM density distribution of genes in different samples revealed that the FPKM distribution between samples in the gestational lactation and non-lactation groups was good overall (Figure 2B). A total of 4197 genes were differentially expressed between the non-lactation and lactation groups during pregnancy. When the threshold values were q < 0.05 and |log2-fold change| ≥ 1, 1974 of these differentially expressed genes were highly expressed in the gestational lactation group, and 2223 differentially expressed genes were highly expressed in the gestational non-lactation group (Figure 2C).

### 3.4. GO and KEGG Pathway Enrichment Analysis of DEGs

**Figure 3:**
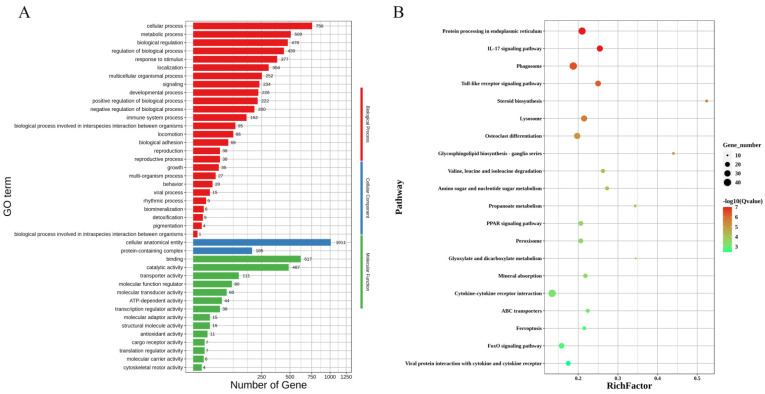
An enrichment analysis of highly expressed genes in the lactating group. (**A**) A GO enrichment analysis of the up-regulated genes in the lactating group. (**B**) A KEGG pathway enrichment analysis of the up-regulated genes in the lactating group.

To obtain a more complete picture of the DEGs, we performed a GO functional enrichment analysis using DAVID. The GO enrichment analysis of highly expressed genes in the lactation group during pregnancy revealed that 42 GO terms were significantly enriched. There were 26 annotations at the biological process level, mainly including the cellular process, metabolic process, developmental process, and reproduction. At the cellular component level, the main annotation was the protein-containing complex. At the molecular function level, there were 14 annotations, mainly including binding, catalytic activity, and transporter activity. The GO annotations of the differentially expressed genes are shown in Figure 3A. The KEGG pathway enrichment analysis revealed that 20 pathways were significantly enriched (*p* < 0.05), including protein processing in the endoplasmic reticulum, the Toll-like receptor signaling pathway, steroid biosynthesis, cytokine–cytokine receptor interaction, amino sugar and nucleotide sugar metabolism, and the PPAR signaling pathway. The pathway annotations of some differentially expressed genes are listed in Figure 3B.

**Figure 4:**
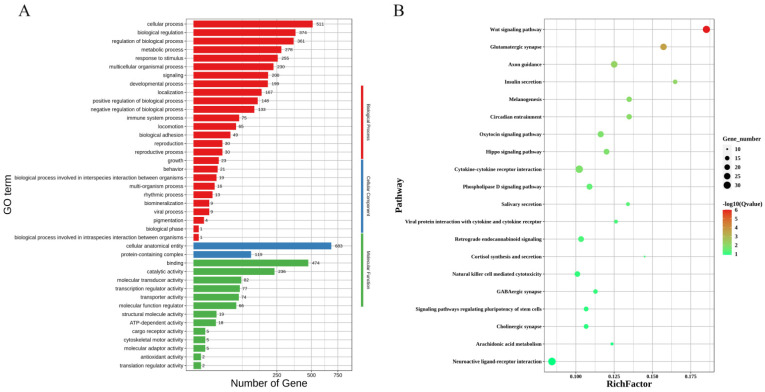
An enrichment analysis of the highly expressed genes in the non-lactating group. (**A**) A GO analysis of the upregulated genes in the non-lactating group. (**B**) A KEGG pathway enrichment analysis of the upregulated genes in the non-lactating group.

The GO enrichment analysis of the highly expressed genes in the non-lactation group during pregnancy revealed 41 GO entries at significant levels. There were 26 entries at the biological process level, including the regulation of the biological process, multicellular organismal process, and signaling; the cellular component level was mainly annotated to protein-containing complexes; and there were 13 entries at the molecular function level, including transcription regulator activity and molecular function regulator (Figure 4A). The KEGG pathway enrichment analysis revealed significant enrichment of the WNT signaling, insulin secretion, arachidonic acid metabolism, and oxytocin signaling pathways (Figure 4B). The different metabolic pathways in these results reveal differences in the metabolic activity between the two groups.

### 3.5. Gene Expression Patterns

**Figure 5:**
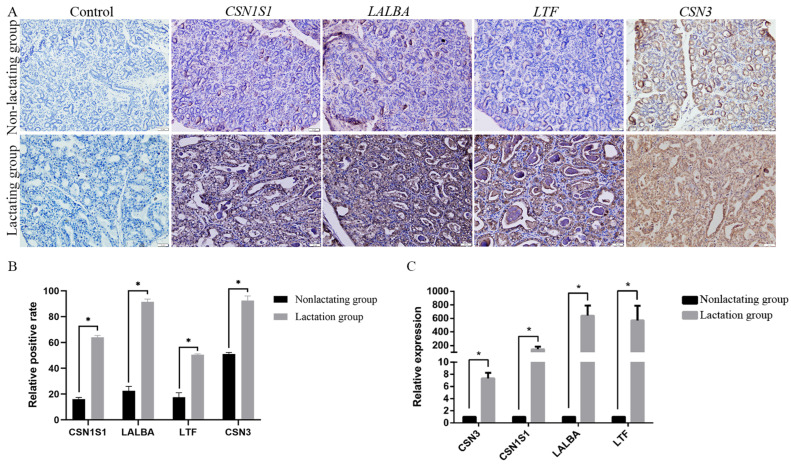
(**A**) Immunohistochemistry of *CSN3*, *CSN1S1*, *LALBA*, and *LTFCSN3* proteins in mammary tissue of lactating pregnant mares. (**B**) Quantitative statistics for (**A**). (**C**) qPCR was used to detect expressions of *CSN3*, *CSN1S1*, *LALBA*, and *LTF* genes in equine mammary tissues. * indicates *p* < 0.05. Data are presented as mean ± SD (n = 3).

To investigate the differences in the gene expression patterns between the lactation and non-lactation groups during pregnancy, we analyzed the genes that were differentially expressed between the lactation and non-lactation groups and selected genes related to the genes encoding the milk proteins α-s1-casein (*CSN1S1*) and k-casein (*CSN3*), whey proteins (*LALBA*), and the lactation event-related gene lactoferrin (*LTF*) in the lactation group for a fluorescence quantitative PCR analysis to confirm the accuracy of the transcriptome sequencing data. Overall, the gene expression patterns in the mammary tissues were consistent with the results of the transcriptome analysis, with *CSN3*, *CSN1S1*, *LALBA*, and *LTF* showing significantly greater mRNA expression in the lactation group than in the non-lactation group (Figure 5B). In addition, the expressions of *CSN3*, *CSN1S1*, *LALBA*, and *LTF* in equine mammary tissues were examined using immunohistochemistry, and the results confirm the RNA-seq and qPCR results, indicating that the *CSN3*, *CSN1S1*, *LALBA*, and *LTF* proteins were highly expressed in the lactation group during pregnancy (Figure 5A,C).

**Figure 6:**
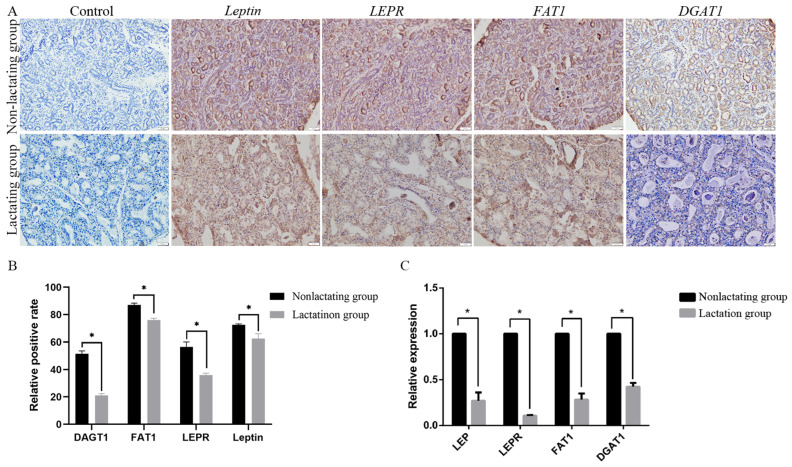
(**A**) Immunohistochemistry of *CSN3*, *CSN1S1*, *LALBA*, and *LTFCSN3* proteins in mammary tissue of nonlactating pregnant mares. (**B**) Quantitative statistics for (**A**). (**C**) qPCR was used to detect expression of *LEP*, *LEFR*, *FAT1*, and *DGAT1* genes in equine mammary tissues. * indicates *p* < 0.05. Data are presented as mean ± SD (n = 3).

In contrast, the genes related to lipid metabolic process diacylglycerol O-acyltransferase 1 (*DGAT1*), leptin (*LEP*), and leptin receptor (*LEPR*) and a gene closely related to lactation events, tyrosine protein kinase (JAK1), were selected from the non-lactation group for qPCR to confirm the RNA-seq results. The results show that the gene expression patterns in the mammary tissues were consistent with the results of the transcriptome analysis, and the mRNA expression levels of *LEP*, *LEPR*, *FAT1*, and *DGAT1* were significantly greater in the non-lactation group than in the lactation group (Figure 6B). The subsequent immunohistochemical results also confirm the consistency of *LEP*, *LEPR*, *FAT1*, and *DGAT1* expression in the equine mammary tissues with the RNA-seq and qPCR results, indicating that the *LEP*, *LEPR*, *FAT1*, and *DGAT1* proteins were highly expressed in the non-lactation group during pregnancy (Figure 6A,C).

## 4. Discussion

Lactation is a process by which milk is secreted by the mammary glands of female mammals and is promoted by a variety of hormones. Moreover, various genes and regulatory molecules are expressed at different stages of lactation and play crucial roles in lactation regulation. To improve the milk production performance of mares, an in-depth understanding of the molecular biology of the lactating mammary gland, such as the expression and regulatory mechanisms of lactation-related genes, is essential.

Transcriptome sequencing of mammary tissue from lactating and non-lactating pregnant mares revealed that 4197 genes were differentially expressed. Among these genes, 1974 were highly expressed in the gestational lactation group, and 2223 differentially expressed genes were highly expressed in the gestational non-lactation group (Figure 2C). Multiple GO classifications and KEGG pathways were found to be involved. The genes highly expressed in the lactation group included *CSN3*, *CSN1S1*, *LALBA*, and *LTF*. Among them, *CSN1S1* and *CSN3* are genes involved in the synthesis of casein, an important protein that has significant effects on milk fat and protein percentage; it has also been demonstrated that casein is closely related to milk production performance in horses [18]. Its deficiency slows the transportation of other casein proteins in goats [19]. In goats, multiple mutations with causal effects on the milk protein and casein contents, cheese yield, and flavor have been identified in the *CSN1S1* gene [20]. The *CSN1S1* genotype was significantly associated with the rennet coagulation time, curd firming time, and curd firmness in Sarda sheep [21]. In cows, the effects of the *CSN1S1* genotype on the protein, casein, and whey protein contents are conflicting, with some suggesting that the BB type is better [22]. In contrast, some suggest that the BC type is better, and others indicate that neither has an effect [22,23]. However, milk from the C variant has a slightly lower fat content [24]. Furthermore, *CSN1S1* polymorphisms are associated with the total milk protein content in the Polish Primitive Horse [25]. Some researchers have investigated the differences in the mRNA expression of milk fat in Kazakh horses with different milk yields, and the results show that the gene expression values of *LALBA*, *CSN1S1*, and *CNS3* were higher in the high-yield group than in the low-yield group, and their DEGs were analyzed by KEGG pathway enrichment, and the amino sugar and nucleotide sugar metabolism pathways were highly enriched, which is a result that is consistent with our research result [18].

The primary function of *CSN3* is to stabilize casein micelles, and enzymatic cleavage of this protein initiates coagulation. In mice, the knockout of the *CSN3* gene, although it does not affect the expression of other milk proteins, leads to the destabilization of casein granules in mammary gland alveoli and reduces lactation in mammals [26]. In cattle, variants of the *CSN3* gene are consistently correlated with the curdling time, milk protein content, and cheese yield [20,27]. In dairy goats, *CSN3* expression was significantly and positively correlated with the protein content of milk, but the correlations with the milk yield and fat content were not significant [28]. Consistent with these findings, there were also significant correlations between *CSN3* genotypes and the milk protein and casein contents in Sarda sheep [21]. A study on the effects of the *CSN3* polymorphism on the milk yield, fat content, casein content, and lactose content revealed that the *CSN3* BB type had a high milk yield and casein content [29].

Similar to casein genes, *LALBA* was highly expressed in the mammary tissue of mares in the lactation group during pregnancy. *LALBA* is part of the lactose synthase complex, which is involved in the formation of lactose and promotes milk production and secretion and is a plausible candidate gene for milk yield traits [30]. During the lactation cycle in humans, cows, and mice, *LALBA* is expressed at levels similar to those of other milk protein genes, and its expression levels increase dramatically with the onset of lactation [31]. Bleck et al. [32] reported that the concentration of *LALBA* in milk was positively correlated with the amounts of protein, fat, and lactose in the milk. Sheep *LALBA* gene polymorphisms were highly correlated with the milk protein and fat contents in the Churra breed [33]. Moreover, *LALBA* gene polymorphisms are significantly associated with milk production performance, and mutations in this gene affect the milk production performance of cows [34]. However, the effects of this gene and the genotype of *CSN1S1* on the milk process quality were not significant [23]. Moreover, the regulatory variants of the *LALBA* gene seemed to have no significant effect on the milk composition or coagulation properties of Sarda sheep [21]. This gene may be associated with a high lactose content in equine milk and has a crucial role in equine lactation.

Lactoferrin (*LTF*) is a multifunctional glycoprotein widely found in human and mammalian milk. It is involved in immunomodulation and has a variety of functions, such as antibacterial, anti-inflammatory, antiviral, and antioxidant activities. The immunomodulatory role of *LTF* lies in its ability to regulate innate and adaptive immunity [35]. It has been shown that the breed of horse and the duration of lactation have significant effects on the amount of *LTF* in milk, and the polymorphisms of the *LTF* gene are breed-specific. The highest level of *LTF* was found in the Polish Warmblood Horse compared with the Polish Primitive Horse and the Polish Coldblood Horse, and the concentration of *LTF* in horse milk was the highest at the fifth week of postnatal life until it decreased significantly at the tenth week of postnatal life [36]. Consistent with these findings, the expression of the porcine *LTF* gene was found to decrease gradually with lactation, indicating that it is closely related to mammary gland development [37]. In addition, Yang Kun et al. [38] reported that the average daily milk yield differed among Ili horses with different genotypes of *LTF* (GG > GA > AA). Similarly, in Chinese Holstein cows, polymorphisms in the *LTF* gene were significantly correlated with milk production shapes, such as the average daily milk yield and milk fat percentage [39].

In addition, the expression profiles of lipid metabolism-related genes were significantly different between the two groups. Among them, *DGAT1* is one of the many proteins that make up the TAG synthesis pathway [40]. This gene plays an important role in the formation of milk fat in mammary epithelial cells, and its polymorphism is closely related to the synthesis of milk fat, which is an important quantitatively inherited trait in the lactation process [41]. Studies have shown that the *DGAT1* gene is critical for mammary gland development and milk synthesis in mice [42]. Additionally, *DGAT1* plays a key role in increasing milk TAG [43]. Strucken et al. [44] reported that *DGAT1* has highly significant effects on milk yield and milk fat at the peak of the laying season. In contrast, Bionaz M [45] indicated that *DGAT1* is less important for overall milk fat synthesis than other genes involved in TAG synthesis, but relative mRNA abundance is moderately upregulated during early lactation.

Leptin, encoded and regulated by the *LEP* gene, is a protein hormone secreted mainly by white fat, and its activity is mediated by the transmembrane receptor OB-R, which is encoded and regulated by *LEPR* [46]. Leptin can be synthesized and secreted by mammary epithelial cells, and its absence leads to a significant weakening of mammary duct branching in mice, which affects their development [47,48]. Leptin can increase the expression of the β-casein gene in the mammary tissue of dairy goats during lactation [49]. Wang Nan [50] reported that leptin activated the PI3K–AKT–mTOR signaling pathway and upregulated the expression levels of the milk fat synthesis-related genes *ACC*, *FASN*, *DGAT1*, and *SREBP1*, thereby increasing milk fat synthesis. In addition, *LEP* and *LEPR* gene mutations inhibit lipid metabolism [51]. Leptin knockdown in the transverse colon of mice resulted in a decreased expression of genes related to lipid synthesis [52]. In the present study, we found that lower expressions of *DGAT1*, *LEP*, and *LEPR* during the lactation stage in horses inhibited milk fat synthesis, which may be responsible for the lower fat content in equine milk than in cow milk.

Regarding the study of different milk production in horses, in addition to the study of mRNAs of horse milk lipids, there are laboratories that have used RNA-Seq technology to study the differentially expressed miRNAs between the milk production phenotypes of high- and low-producing Kazakh horses, and a total of 83 differentially expressed miRNA and 2415 genes have been identified, which are enriched for mammary gland development, mammary gland morphogenesis, tissue development, the PI3K-Akt signaling pathway, insulin signaling pathway, Wnt signaling pathway, and TGF-β signaling pathway. The insulin signaling pathway and the Wnt signaling pathway were also enriched in our analysis, indicating the importance of this pathway in equine lactation [53].

There was also a report that genome-wide DNA methylation profiles of high- and low-milk-yielding Kazakh equine blood were investigated using deep whole-genome bisulfite sequencing, and 27,172 differentially methylated regions and 8268 differentially methylated region-associated genes (DMGs) were identified, and their functional enrichment analysis of overlapping differentially methylated genes in combination with WGBS-seq and RNA-seq data revealed that nine DMGs in the AGE-RAGE and PI3K-Akt signaling pathways, including GJA1, RAC1, LTBP1, TIAM1, SERPINB6, DGKG, IL7, FN1, and COL1A1, were the ones that might play a decisive role in milk production in Kazakh horses [54].

In our study, through an enrichment analysis of differentially expressed genes in the lactation and non-lactation groups, we screened genes encoding milk protein-related genes (*CSN1S1*, *CSN3*, and *LALBA*), genes related to lipid metabolism processes (*DGAT1*, *LEP*, and *LEPR*), and genes that are closely related to lactation events (*FAT1* and *LTF*), which can be used as candidate genes to select for mare lactation traits. In addition, there were differences in metabolic activities between the two groups, e.g., amino sugar and nucleotide sugar metabolism were highly enriched in the lactation group, whereas arachidonic acid metabolism was highly enriched in the non-lactation group. Furthermore, the PPAR signaling pathway was highly enriched in the gestational lactation group, and it may also play a key role in mare lactation.

## 5. Conclusions

In summary, we analyzed the mammary glands’ transcriptome expression differences between lactating and non-lactating mares in the second month of gestation using RNA-seq and obtained a total of 4197 DEGs, and their functional analyses are of theoretical and practical reference value for the further development of molecular breeding research in dairy horses.

## Data Availability

RNA-seq raw data were deposited in the following NCBI database: BioProjects PRJNA1142614.

## Supplementary Materials

The following supporting information can be downloaded at https://www.mdpi.com/article/10.3390/ani14162319/s1, Supplementary Table S1: Quality control of Mongolian horse mammary RNA-seq data; Supplementary Table S2: The reads were aligned to the reference genome; Supplementary File S1: Equine reference genome information; Supplementary Table S3: qPCR primers for DEG validations; Supplementary Table S4: RNA-seq data statistics.
