# Supplementary material for: A Comparative Analysis of the Gene Expression Profiles in the Mammary Glands of Lactating and Nonlactating Mares at the Second Month of Gestation
Source: Animals (Basel). 2024 Aug 9;14(16):2319. doi: 10.3390/ani14162319 (PMC11350905; doi:10.3390/ani14162319)
Supplement: Supplementary file 1 [file animals-14-02319-s001.zip › Supplementary File S1.pdf]

Equine reference genome information

Genome: GCF_002863925.1_EquCab3.0_genomic.fna

https://www.ncbi.nlm.nih.gov/datasets/genome/GCF_002863925.1/
